# Expert Consensus on Prevention and Treatment of COVID-19 Infection in Patients with Lung Cancer

**DOI:** 10.3779/j.issn.1009-3419.2023.102.12

**Published:** 2023-03-20

**Authors:** Non-small Cell Lung Cancer Expert Committee of Chinese Society of Clinical Oncology, China Medical Education Association

**Keywords:** Corona virus disease 2019, Lung neoplasms, Prevention, Treatment, Expert consensus

## Abstract

Corona virus disease 2019 (COVID-19) infection has become a major public health issue affecting human health. The main goal of epidemic prevention and control at the current stage in China is to “protect people’s health and prevent severe cases”. Patients with lung cancer who receive antitumor therapy have low immunity, and the risk of severe illness and death once infected is much higher than healthy people, so they are vulnerable to COVID-19 infection. At present, less attention has been paid to the prevention and treatment of COVID-19 infection in patients with lung cancer in domestic guidelines and consensus. Based on the published data in China and abroad, we proposed recommendations and formed expert consensus on the vaccination of COVID-19, the use of neutralizing antibodies and small molecule antiviral drugs for patients with lung cancer, for physician’s reference.

## 1 Introduction

Lung cancer is a malignant tumor with high morbidity and mortality in China and other countries around the world. The 5-year survival rate is only 19%^[1]^. Since the rapid spread of Corona virus disease 2019 (COVID-19) infection worldwide, it has become the third cause of death in the United States in 2021^[2]^. Due to the old age of most patients with lung cancer, multiple underlying diseases, immunosuppression caused by the antitumor therapy, repeated visits to hospitals, and other factors, the risk of COVID-19 infection, severe illness, and death of patients with lung cancer is much higher than that of healthy people^[3]^.

Vaccination is still the main effective way to prevent COVID-19 infection and severe illness in patients with lung cancer. However, patients with cancer are often excluded from the clinical trials of vaccine development. The efficacy and safety of vaccination in patients with cancer remain to be studied, and it is unclear how to better play the synergistic effect of prevention and treatment. In addition, the immune function of patients with lung cancer is impaired, the effects of vaccine prevention and treatment need further observation. The goal of prevention and treatment of COVID-19 infection in patients with cancer and vaccination and immunization strategies need to be determined. Based on the clinical practice of COVID-19 infection in patients with lung cancer, considering the relevant data in China and abroad and China’s epidemic prevention and control policies, the Non-small Cell Lung Cancer (NSCLC) Expert Committee of Chinese Society of Clinical Oncology (CSCO) and China Medical Education Association have formed the first expert consensus on prevention and treatment of COVID-19 infection in patients with lung cancer after extensive consultation.

The principles of expert recommendation and evidence rating in this consensus are as follows:

**Recommendation level: A=strong; B=moderate; C=optional. Evidence level: I=1 or more randomized trials without significant limitations; IIa=other randomized trials or subgroup analyses of randomized trials; IIb=non-randomized trials or observational cohort studies; III=expert opinion.**

## 2 Epidemiology of lung cancer during COVID-19

### 2.1 Epidemic trend of COVID-19

According to the World Health Organization (WHO), as of 18 December 2022, the cumulative number of confirmed COVID-19 infection cases worldwide was more than 649 million, with more than 6.6 million people died^[4]^. Data from the United States in 2021 showed that COVID-19 infection has become the “number one killer” among middle-aged and elderly people (≥45 years old) in the United States^[2]^. China is a country with a large population, a large number of vulnerable people, unbalanced regional development, and insufficient medical resources. Affected by virus mutation, spring and winter climate, and prevention and control policy factors, how to ensure the safety of people at high risk of COVID-19 infection is a key and decisive factor in achieving the goal of “protecting people’s health and preventing severe cases”.

### 2.2 Current status of COVID-19 infection in patients with lung cancer

It is estimated that about 870,000 new cases of lung cancer are expected in China (excluding Taiwan province) in 2022, accounting for 18.07% of the total number of new cancers, ranking first among malignant tumors in China^[1]^. The average age of lung patients is 70 years old^[5]^, and most of them are also suffering from smoking-related chronic airway and cardiovascular diseases, etc. Coupled with surgery, chemotherapy, radiotherapy, patients’ immunity is weakened. Compared with the general population, patients with lung cancer have a higher risk of COVID-19 infection and a higher proportion of severe illness and death. A retrospective case-control study in the United States showed that patients with newly diagnosed lung cancer had a significantly increased risk of COVID-19 infection [odds ratio (OR)=7.66; 95%CI: 7.07-8.29; P<0.001]^[6]^. A retrospective analysis in China showed that lung cancer accounted for the highest proportion of patients with cancer infected with COVID-19, at 20.9% (22/105)^[7]^. In 2022, a meta-analysis of 21,257 patients with lung cancer who were infected with COVID-19 showed that the mortality rate of patients with lung cancer was significantly higher than that of patients without cancer [hazard ratio (HR)=2.00; 95%CI: 1.52-2.63; P<0.01], and also higher than that of patients with other tumors (HR=1.91; 95%CI: 1.53-2.39; P<0.01)^[7]^. A retrospective analysis in the United States in 2020 showed that 62% of patients with lung cancer who were infected with COVID-19 required hospitalization, 40% required intensive care unit (ICU) treatment, and the mortality rate was as high as 25%^[8]^.

## 3 COVID-19 vaccination in patients with lung cancer

### 3.1 COVID-19 vaccination in patients with lung cancer before exposure to COVID-19

#### 3.1.1 Full immunization with COVID-19 vaccines in patients with lung cancer

**Consensus 1: Patients with lung cancer, who are susceptible to COVID-19 and at high risk of severe illness and death from the virus, should be vaccinated with priority. It is recommended that patients should be fully assessed and encouraged to receive the full course of vaccination against COVID-19 as early as possible (Recommendation level: A; Evidence level: I).**

Domestic guidelines and consensus recommended that patients with malignant tumors should receive COVID-19 vaccines. The safety of patients with cancer receiving COVID-19 vaccines is comparable to that of the general healthy population^[9,10]^. Patients with cancer can be vaccinated against COVID-19, but the immune response and protective effect are lower, according to the “Technical guidelines for vaccination of COVID-19 (version 1)”. Patients with advanced cancer are not advised to receive COVID-19 vaccines^[9]^. According to “The Chinese expert consensus on issues related to the protection, treatment and management of patients with solid tumors during COVID-19”, the type and degree of common adverse reactions of COVID-19 vaccination in patients with cancer are similar to those in healthy people, mainly manifested as injection site pain, fatigue, fever, chills, headache and myalgia^[10]^.

COVID-19 vaccination in patients with lung cancer can increase the concentration of neutralizing antibodies and reduce the risk of severe infection, and the vaccination is safe. A cohort study of 201 patients with cancer (including 102 patients with lung cancer) showed that the seropositive rate of receptor binding domain (RBD) immunoglobulin G (IgG) in patients with cancer was 81.6% after receiving 2 doses of inactivated vaccines from Sinovac (CoronaVac), no grade 3 or above adverse reactions occurred^[11]^. An observational study of 364 patients with cancer showed that 239 patients (86.9%) were seropositive after 2 doses of inactivated vaccines from Beijing Institute of Biological Products Co. Ltd (BBIBP-CorV); in terms of the safety, pain at the injection site and fever were the most common adverse reactions^[12]^. A total of 719,735 patients were enrolled in the large cohort study in Colombia, of which 9,308 were patients with cancer. The results showed that vaccination with 2 doses of inactivated vaccines from Sinovac could reduce the hospitalization rate of COVID-19 infection (33.1%; 95%CI: 14.5-47.7), admission rate of severe illness (47.2%; 95%CI: 18.5-65.8), and mortality rate (55.7%; 95%CI: 32.5-70.0)^[13]^. The above results indicated that COVID-19 vaccination has a protective effect on patients with lung cancer, and the vaccination is safe.

#### 3.1.2 Booster immunization with COVID-19 vaccines in patients with lung cancer

**Consensus 2: Patients with lung cancer who have completed the full immunization against COVID-19 are revised to receive the first booster immunization after an interval of 3 months; and the second booster immunization after an interval of 6 months after the completion of the first booster immunization (Recommendation level: A; Evidence level: I).**

After the full immunization, the booster immunization can increase the protective effect on patients with cancer and reduce the COVID-19 infection rate, hospitalization rate, and mortality rate. In a retrospective study of 2,578 patients with cancer (including 325 with NSCLC), patients with cancer who received booster immunization (2 doses of inactivated vaccines from Sinovac + 1 dose of BNT162b2 + 3 doses of inactivated vaccines from Sinovac) had a lower rate of COVID-19 infection compared with patients who did not receive booster immunization (83.7% vs 16.3%; P=0.000)^[14]^. A prospective study of 346 patients with breast cancer, 296 with NSCLC, and 32 with small cell lung cancer showed that the third dose of BNT162b2 vaccine (156 patients) significantly reduced the risk of hospitalization or death in patients with breast cancer compared with those without the vaccines (48 patients) (OR=0.30; 95%CI: 0.15- 0.57; P=0.01), while there were no significant changes in patients without the third dose of the vaccine (133 patients) (OR=0.64; 95%CI: 0.3-1.24; P=0.19)^[15]^. A multicenter study of 97 patients with solid tumors (including 12 with NSCLC) and 82 healthy participants who received 2 doses of the vaccines but were seronegative showed a seropositive rate of approximately 30% in patients with solid tumors (71% in the healthy participants) after the third dose of inactivated vaccines from Sinovac, indicating that 3 doses of the vaccines had protective effect in patients with solid tumors^[16]^. Results of a cohort study showed that the serological response of 216 patients with solid tumors (lung cancer: 23.4%) was assessed after the third dose of BNT162b2 vaccine, compared with the first month after the vaccination, the antibody titer of patients with solid tumors decreased by 1.94-fold at 4 months after the vaccination (1,239.63 AU/mL vs 639.42 AU/mL). The estimated half-life of serum IgG in patients with solid tumors was 88 days, and the time for serum antibody to turn negative was 588 days, indicating that patients with cancer needed to receive a second dose of booster immunization^[17]^.

Several domestic and foreign guidelines recommended booster immunization for patients with malignant tumors. According to “The Chinese expert consensus on issues related to the protection, treatment and management of patients with solid tumors during COVID-19 (2022 edition)”, patients with cancer should receive the recommended dose and complete the recommended number of doses^[10]^. According to the United States National Comprehensive Cancer Network (NCCN), most patients with cancer (except those in the perioperative period of major surgery) should complete the full vaccination and the second booster vaccination as early as possible^[18]^.

According to China’s “Work plan for strengthening the vaccination of the elderly against COVID-19”, the interval between the first dose of booster immunization and the full vaccination should be adjusted to more than 3 months, based on the data from real world studies and clinical trials in China and abroad, as well as the actual vaccination of the elderly population in China^[19]^. Therefore, it is recommended that patients with cancer should complete the first booster vaccination at 3 months after the full vaccination.

As for the second booster vaccination, according to China’s “Notice on issuing the implementation plan for the second booster vaccination of COVID-19”, on the basis of the first booster vaccination, the second booster vaccination should be administered in people at high risk of infection, elderly people over 60 years of age, people with more serious underlying diseases, and people with low immunity (including patients with cancer)^[20]^. According to the actual vaccination practice in China, the interval between the second dose and the first dose of booster vaccination should be more than 6 months. Therefore, it is recommended that patients with cancer should complete a second booster vaccination 6 months after the first booster vaccination.

### 3.2 Vaccination of patients with lung cancer after previous COVID-19 infection

**Consensus 3: Patients with lung cancer who have previously been infected with COVID-19 are recommended to receive the vaccine after 6 months of recovery (Recommendation level: B; Evidence level: IIb).**

On 20 December 2022, the Joint Prevention and Control Mechanism of the State Council stated at the press conference on medical services for key populations that, if it was confirmed by nucleic acid test or antigen test that the patient had recently been infected with COVID-19, the risk of re-infection within 3 to 6 months was low, and it was not recommended to vaccinate the infected people in a short period of time. According to the current vaccination recommendations, the vaccine can be given 6 months after COVID-19 infection.

The mixed immune response produced by the infection and vaccination is superior to that produced by the infection alone or vaccination alone^[21]^. A cohort study in 2021 included 63 former COVID-19 patients, 41% of whom were vaccinated against COVID-19. The results showed that RBD antibody reactivity, neutralizing activity, and the number of RBD-specific memory B cells in unvaccinated recovered patients remained relatively stable between 6 and 12 months after the infection, while the antibody neutralizing activity in vaccinated recovered patients was further improved by about 50-fold^[22]^. Patients with cancer with impaired immune function are still at the risk of being re-infected with COVID-19. It is recommended that patients with lung cancer should be actively vaccinated 6 months after recovery.

### 3.3 COVID-19 vaccination in patients with lung cancer during antitumor therapy

#### 3.3.1 COVID-19 vaccination during surgery

**Consensus 4: For patients with lung cancer requiring surgery, it is recommended that the vaccination date should be ±2 weeks from the surgery date. If the patient receives the COVID-19 vaccine and experiences vaccine-related adverse reactions, surgery for the lung cancer should be considered after the vaccine-related adverse reactions completely resolve, except for emergency surgery (Recommendation level: C; Evidence level: III).**

Patients undergoing lung cancer surgery are a priority group for COVID-19 vaccination. A study evaluated the population in priority for vaccination through modeling. The study was stratified by whether they underwent surgery (general population, cancer surgery, non-cancer surgery) and age (18-49 years, 50-69 years, ≥70 years), with the primary endpoint being the number needed to vaccinate (NNV) to prevent 1 death caused by COVID-19 infection within 1 year. The results showed that patients aged ≥70 years requiring cancer surgery had the greatest benefit from vaccination compared with the general population [NNV of 1,840 (1,196-3,066)], with NNV of 351 (196-816). Preoperative vaccination of patients requiring elective surgery over the general population could prevent an additional 58,687 (20,177-115,007) deaths related to COVID-19 infection within 1 year^[23]^.

Vaccination against COVID-19 may lead to adverse reactions such as fever. To accurately distinguish vaccine-related adverse reactions from surgical complications, it is recommended that the vaccination should be more than 2 weeks apart from the date of surgery.

#### 3.3.2 COVID-19 vaccination during radiotherapy/chemotherapy

**Consensus 5: During the radiotherapy/chemotherapy, the immune function decreases so that the protective efficacy of vaccine is decreased. Patients with lung cancer undergoing radiotherapy/chemotherapy are advised to postpone COVID-19 vaccination. Patients who discontinue the radiotherapy/chemotherapy for more than 1 month are advised to receive COVID-19 vaccines (Recommendation level: B; Evidence level: IIb).**

The antibody response rate of patients with lung cancer vaccinated during the chemotherapy is lower and the protective efficacy of the vaccine decreases. A study of 201 patients with cancer (102 of whom had lung cancer) and 97 non-cancer patients showed that the antibody response to the vaccine was lower in patients with cancer who were receiving chemotherapy after 2 doses of inactivated vaccines from Sinovac (OR=0.303; 95%CI: 0.123-0.750; P=0.010) ^[11]^. A study of 776 patients with cancer (23.6% of whom had lung cancer) and 715 non-cancer patients showed that seropositive rates were significantly lower in the chemotherapeutic group than in the non-chemotherapeutic group after receiving inactivated vaccines from Sinovac (78.6% vs 91.1%; P<0.001)^[24]^. In addition, a meta-analysis of 39 studies, including 11,075 patients with cancer, showed that serum response was significantly lower in patients treated with antitumor therapy than in the untreated group (OR=2.55; 95%CI: 2.04-3.18), and the seroconversion rate of patients with chemotherapy was lower than that of the group without chemotherapy (OR=3.04; 95%CI: 2.28-4.05)^[25]^.

The Joint Prevention and Control Mechanism of the State Council stated in the “Work plan for strengthening the vaccination of the elderly against COVID-19” that patients with severe chronic diseases that are in the acute stage, such as patients with cancer undergoing chemotherapy, should postpone the vaccination^[19]^. According to “Expert advice on novel coronavirus vaccination in patients with malignant tumors”, patients undergoing chemotherapy are not advised to receive COVID-19 vaccines, while those who have completed radiotherapy and chemotherapy for more than 1 month are advised to receive COVID-19 vaccines^[26]^. According to “The Chinese expert consensus on issues related to the protection, treatment and management of patients with solid tumors during COVID-19”, patients with cancer should be vaccinated at least 2 weeks before chemotherapy or 1 to 2 weeks after the chemotherapy^[10]^.

No large sample clinical studies have been conducted to investigate the safety and efficacy of COVID-19 vaccines in patients with lung cancer receiving radiotherapy. A case report showed that an NSCLC patient who received 2 doses of COVID-19 vaccines during the radiotherapy developed acute radiation pneumonia^[27]^. Due to insufficient data on the safety of vaccination during the radiotherapy, it is recommended to postpone the vaccination during the radiotherapy. According to “The Chinese expert consensus on issues related to the protection, treatment and management of patients with solid tumors during COVID-19”, in the absence of sufficient clinical data at this stage, patients with short-course radiotherapy can be vaccinated after the course of treatment, or at any stage of radiotherapy, taking into account the risk of COVID-19 infection and the patient’s willingness^[10]^. According to “Expert advice on novel coronavirus vaccination in patients with malignant tumors”, it is not recommended for patients undergoing radiotherapy, but can be vaccinated against COVID-19 after the radiotherapy^[26]^.

#### 3.3.3 COVID-19 vaccination during immune checkpoint inhibitors (ICIs) therapy

**Consensus 6: During the treatment of ICIs, a thorough evaluated should be made, and patients should be vaccination with caution (Recommendation level: B; Evidence level: III).**

Several studies showed that COVID-19 vaccination to patients with cancer who are receiving ICIs is safe in the short term, but the long-term safety needs further assessment. A study of 454 patients with cancer (including 157 with NSCLC) showed that receiving programmed cell death 1 (PD-1) inhibitors did not affect the serological response after the vaccination with inactivated vaccines (68.1% vs 71.3%)^[28]^. A real-world study of 2,048 patients with cancer (including 722 with lung cancer) receiving PD-1 inhibitors showed that compared with the unvaccinated group (530 patients), the group vaccinated with inactivated vaccines from Beijing Institute of Biological Products Co. Ltd (1,518 patients) neither increased the serious adverse reactions of PD-1 inhibitors nor reduced the efficacy of PD-1 inhibitors^[29]^. A meta-analysis of 39 studies involving 11,075 patients with cancer showed that the serological response of patients with cancer receiving immunotherapy (mainly including chimeric antigen receptor T cell therapy and ICIs) had no statistically significant difference compared with untreated patients (OR=1.23; 95%CI: 0.85-1.76; P=0.27), suggesting that the use of ICIs may not affect the protective effect of COVID-19 vaccines^[25]^.

Lymphopenia, T cell depletion caused by high checkpoint expression, and inflammatory cytokine storm are the three most important immunological manifestations associated with poor outcome of COVID-19. However, ICIs may potentially reactivate the depleted T cells, thus aggravating the inflammatory cytokine storm caused by COVID-19 and leading to organ damage^[30]^. According to “Expert advice on novel coronavirus vaccination in patients with malignant tumors”, vaccination is not recommended for patients undergoing immunotherapy, as there is a risk of an over-strong immune response after vaccination, leading to or aggravating immune-related adverse reactions^[26]^.

Therefore, COVID-19 vaccination during ICIs treatment should be considered with caution after a thorough assessment, weighing the risks against the benefits.

## 4 Prevention of COVID-19 using long-acting neutralizing antibodies in patients with lung cancer

### 4.1 Prevention of COVID-19 using neutralizing antibodies in patients with lung cancer

**Consensus 7: Patients with lung cancer are vulnerable population, whose immune response rate and neutralizing antibody titers after the vaccination are lower than those of the general healthy population. It is recommended that patients with lung cancer use long-acting neutralizing antibody against COVID-19 (Tixagevimab/Cilgavimab, 600 mg, intramuscularly, once every 6 months) as a means of pre-exposure prevention to reduce the risk of COVID-19 infection, severe illness, and death. After the vaccination, it is recommended to use Tixagevimab/Cilgavimab after an interval of 2 weeks; after Tixagevimab/Cilgavimab injection, the vaccine can be administered without an interval (Recommendation level: B; Evidence level: I).**

Even if patients with lung cancer are vaccinated, their immune response and neutralizing antibody titers are lower than those of the general healthy population. They are still at high risk of COVID-19 infection. A real-world retrospective study with the largest sample size of COVID-19 infection cases and control group cases to date conducted in the United States showed that the risk of breakthrough infection in patients with solid tumors was 1.12-folder higher than that of non-cancer patients even after full vaccination (OR=1.12; 95%CI: 1.01-1.23); the severity rate was 1.33-fold higher than that of non-cancer patients (OR=1.33; 95%CI: 1.09-1.62)^[31]^. In addition, the antibody titers were significantly lower in patients with lung cancer than in general healthy population after the vaccination. A study^[32]^ assessing the antibody response of 82 patients with NSCLC and 53 healthy participants after receiving the mRNA booster (the third shot) showed that the neutralizing antibody titer in patients with NSCLC at 1 month after 2 doses of mRNA vaccines was 7 times lower than those in healthy participants (P≤0.0001); 2-4 months after the third dose of the booster injection, the neutralizing antibody titer of the original and Omicron strains decreased by 5 and 7 times, respectively.

During the active antitumor therapy, the immune function of patients with lung cancer decreases and the protective effect of vaccine decreases. The protective effect of COVID-19 vaccine significantly reduces during the radiotherapy/chemotherapy in patients with cancer. A cohort study in United Kingdom of 230,666 patients with cancer who had received 3 doses of COVID-19 vaccines showed an increase in both breakthrough and symptomatic rates of infection in patients who received systemic antitumor therapy or radiotherapy within 1 year, compared with those who did not^[33]^. Another study including 201 patients with cancer (99 with breast cancer, 102 with lung cancer) and 97 non-cancer patients who had completed 2 doses of vaccines showed that patients with cancer who were receiving active chemotherapy had lower antibody responses to the vaccines (OR=0.303; 95%CI: 0.123-0.750; P=0.010)^[11]^. In addition, there are also some patients with lung cancer in China have never received COVID-19 vaccines. Therefore, it is particularly necessary to use neutralizing antibodies as passive immune protection for patients with lung cancer who are undergoing radiotherapy/chemotherapy.

Monoclonal antibody drugs designed for COVID-19 can specifically bind to viral particles and have neutralizing capabilities, thus helping to clear and reduce the viral load in the body. Therefore, neutralizing antibodies can provide susceptible individuals with antibodies that cannot be produced by the body itself to neutralize the virus, offering hope for patients with lung cancer to prevent COVID-19 infection. Patients with suppressed immune function (eg, patients with solid and hematological tumors, patients with primary and acquired immunodeficiency, and patients with solid organ and hematopoietic stem cell transplantation) may be passively immunized with long-acting neutralizing antibodies to achieve a higher level of immune protection.

Tixagevimab/Cilgavimab is the only drug approved in the world for both prevention and treatment of COVID-19. It is a cocktail of neutralizing antibodies targeting 2 different regions of the spike protein of the virus, enhancing the ability to neutralize the virus and new mutant strains^[34]^. The PROVENT study, a phase III clinical study of Tixagevimab/Cilgavimab, included 5,197 adults worldwide who had an inadequate response to the vaccine or were at high risk of COVID-19 infection, 7.7% of whom had malignant tumors and were randomized to receive either a single dose of Tixagevimab/Cilgavimab (n=3,460) or placebo (n=1,737). The results showed that Tixagevimab/Cilgavimab significantly reduced the relative risk of symptomatic COVID-19 infection by 83% (P<0.001), with a protective effect of ≥6 months^[35]^. In terms of safety, most of the adverse event within 15 months after a single dose were mild or moderate and comparable to those in the placebo group.

Multiple meta-analyses showed that Tixagevimab/Cilgavimab had good protective effect against COVID-19 in the real world. A recent meta-analysis in 2022 of 30 studies with 27,932 participants who were at high risk of COVID-19 infection (66.6% of whom were immunocompromised) showed that Tixagevimab/Cilgavimab reduced the hospitalization rate associated with COVID-19 infection compared with the control group (0.54% vs 1.2%; P=0.27), ICU admission rate (0.6% vs 5.2%; P=0.68), mortality rate (0.2% vs 1.2%; P=0.67), proportion of patients requiring oxygen therapy (8% vs 41.2%; P=0.37), reverse transcription-polymerase chain reaction (RT-PCR) positive rate (2.1% vs 5.8%; P=0.01), severe COVID-19 pneumonia (0% vs 0.5%; P=0.79), and symptomatic COVID-19 pneumonia (1.8% vs 6%; P=0.22)^[36]^. A systematic review in United Kingdom involved 24,773 immunocompromised patients from 17 studies, the clinical efficacy of prophylactic Tixagevimab/Cilgavimab therapy for breakthrough COVID-19 infection, hospitalizations, and ICU admissions were 40.47%, 69.23%, and 87.89%, respectively (all P<0.0001). The total clinical efficacy for preventing all-cause mortality and mortality associated with COVID-19 infection were 81.29% (P<0.0001) and 86.36% (P=0.0351), respectively^[37]^.

In multiple real-world studies during the Omicrone epidemic, Tixagevimab/Cilgavimab significantly reduced the rate of COVID-19 infection and hospitalization in cancer populations receiving radiotherapy/chemotherapy. In a retrospective analysis of electronic medical records of the veterans from the United States during the Omicrone epidemic, preference score matched 1,733 patients who received ≥1 dose of Tixagevimab/Cilgavimab and 6,354 patients in the control group, 34% of whom had a malignant tumor. Tixagevimab/Cilgavimab significantly reduced the composite endpoint of COVID-19 (including COVID-19 infection, hospitalization, and all-cause mortality) by 69% [17/1,733 (1.0%) vs 206/6,354 (3.2%); HR=0.31; 95%CI: 0.18-0.53], the risk of COVID-19 infection by 66% (HR=0.34; 95%CI: 0.13-0.87), the risk of hospitalization associated with COVID-19 by 87% (HR=0.13, 95%CI: 0.02-0.99), and the risk of all-cause mortality by 64% (HR=0.36; 95%CI: 0.18-0.73)^[38]^. Another real-world study of 1,295 immunocompromised patients, 15.1% of whom were receiving chemotherapy, showed that Tixagevimab/Cilgavimab significantly reduced the rate of hospitalization associated with COVID-19 by 80.2% [risk ratio (RR)=0.20; 95%CI: 0.09-0.45; P<0.001]^[39]^.

In March 2022, the European Union approved Tixagevimab/Cilgavimab for the pre-exposure prophylaxis against COVID-19 in adults and adolescents over 12 years of age (body weight >40 kg). On 8 December 2021, the Food and Drug Administration (FDA) approved the Emergency Use Authorization for Tixagevimab/Cilgavimab as a pre-exposure prophylaxis measure for moderately to severely immunocompromised patients or patients who do not receive COVID-19 vaccines due to a history of severe adverse reactions^[40]^. The recommended dose is 300 mg of Tixagevimab and 300 mg of Cilgavimab administered by continuous intramuscular injection, and repeated every 6 months to ensure lasting protective effect.

The United States NCCN guidelines recommended that immunocompromised patients with cancer, including those receiving antitumor therapy, were prioritized for the use of long-acting neutralizing antibody of Tixagevimab/Cilgavimab, as a preventive measure against COVID-19. The vaccine can be administered immediately after the administration of Tixagevimab/Cilgavimab. For those who receive the vaccine first, it is recommended to use Tixagevimab/Cilgavimab 2 weeks later to avoid affecting the vaccine-induced immune response. Tixagevimab/Cilgavimab is not a substitute for COVID-19 vaccine^[41]^.

### 4.2 The use of neutralizing antibody after the recovery from COVID-19 infection in patients with lung cancer

**Consensus 8: Patients with lung cancer who have recovered from previous infection with COVID-19 can use long-acting neutralizing antibody (Tixagevimab/Cilgavimab, 600 mg, intramuscularly) after 3 to 6 months of recovery to reduce the rate of COVID-19 re-infection and hospitalization (Recommendation level: B; Evidence level: IIb).**

Given that the virus is constantly mutating, patients with lung cancer who have already been infected with COVID-19 are still at risk of being re-infected with the mutated strains. Clinical studies involving previously infected people showed that the use of neutralizing antibodies can reduce the risk of re-infection. In an Israeli retrospective analysis during the Omicrone epidemic, 5,124 immunocompromised patients over the age of 12 years were included, 825 patients receiving Tixagevimab/Cilgavimab tended to match 4,299 untreated patients, of whom 64% in the treatment group had cancer and 20.7% had previous COVID-19 infection. The results showed that compared with the untreated group, the Tixagevimab/Cilgavimab treatment group significantly reduced the risk of COVID-19 infection by 49% (3.5% vs 7.2%; P<0.001; OR=0.51; 95%CI: 0.30-0.84), hospitalization rate for COVID-19 infection (0.1% vs 0.6%; P=0.07) and death by 92% (0% vs 0.9%; P=0.005; OR=0.08; 95%CI: 0.01-0.54)^[42]^. A retrospective cohort study in France included 1,112 immunocompromised patients who had received Tixagevimab/Cilgavimab, of whom 9.6% being previously infected with COVID-19. The results showed that Tixagevimab/Cilgavimab reduced the rate of COVID-19 related hospitalizations by 80.2%^[43]^. Neutralizing antibody levels rapidly decline in humans 3-6 months after COVID-19 infection, thus, it is recommended that patients with lung cancer should be treated with Tixagevimab/Cilgavimab at this time point.

## 5 Daily prevention of COVID-19 infection in patients with lung cancer

**Consensus 9: It is recommended that patients with lung cancer should insist on wearing masks, washing hands frequently, maintaining social distance, and taking good personal protection (Recommendation level: A; Evidence level: I).**

### 5.1 Precautions for daily protection of patients with lung cancer

Patients with lung cancer should maintain good personal and environmental hygiene, balanced nutrition, moderate exercise, adequate rest, and avoid excessive fatigue. Patients with lung cancer should improve health literacy and develop hygiene habits and lifestyles such as “one-meter line”, frequent hand washing, wearing masks, and using serving chopsticks. Patients should keep the room well ventilated and have scientific personal protection. Patients with fever and respiratory symptoms should go to the clinic for medical treatment timely^[44]^. At the same time, family members and long-term caregivers of patients with lung cancer are advised to be vaccinated against COVID-19 to reduce the risk of transmission of the virus. The blood gas barrier is destroyed, the respiratory mucosal immunity and basic immune function of patients with lung cancer are impaired, and about 50% of patients with lung cancer are complicated with chronic obstructive pulmonary disease (COPD)^[45,46]^. For patients with COPD, it is recommended to use Staphylococcus and Neisseria tablets (also known as tracheitis Junmiao tablets, Qi tablets) and bacterial lysates to improve innate immunity and respiratory mucosal immunity, reduce repeated respiratory infections, and reduce the severity and frequency of acute exacerbation of COPD. Staphylococcus and Neisseria tablets are produced after the inactivation of three symbiotic human bacteria, including Staphylococcus albus, Neisserkatascus, and Bacillus subtilis, which are bacteria-derived immunomodulators which activate the non-specific immune system of the digestive tract after oral administration and produce specific antibodies (IgA) in the respiratory tract to enhance respiratory specific immune function^[47⇓-49]^. Bacterial lysates are mainly lyophilized dissolved products of eight common respiratory tract infection bacteria. Clinical studies showed that the combination of bacterial lysates on the basis of conventional treatment can effectively reduce the number of acute exacerbations in patients with COPD, shorten the length of hospital stay and the duration of antibiotic use during the acute exacerbation of COPD, improve the clinical symptoms, and help to improve the lung function and immune function of patients^[50⇓-52]^. Immunomodulators, such as thymalfasin, can enhance immune response to vaccines in patients with lung cancer with suppressed immune function. For patients with hypoxemia and lymphopenia caused by COVID-19 infection, conventional treatment combined with thymalfasin for 5 consecutive days can significantly increase CD4^+^ T cell count and accelerate clinical recovery with good safety^[53]^.

### 5.2 Diagnosis and treatment for patients with lung cancer

During the epidemic period, patients with lung cancer who have relatively stable diseases should minimize the number of visits to the hospital, and can choose the 24-hour online consultation service of regular hospitals to determine the appropriate time for review. For those who need surgical treatment, it is recommended to fully communicate with physicians, fully evaluate the patient’s situation and surgical risks, to determine whether to undergo the surgery, or strengthen protection during surgery, to ensure surgical safety and avoid COVID-19 infection. Patients in the antitumor treatment period have lower immune function and are more susceptible. Protective measures should be further strengthened. If the disease progresses rapidly or other abnormalities occur, patients should go to the hospital as early as possible^[54]^.

## 6 Treatment of COVID-19 infection in patients with lung cancer

### 6.1 Antiviral therapy

**Consensus 10: Patients with lung cancer who infected with COVID-19 should use antiviral drugs (such as antiviral small molecule drugs and COVID-19 neutralizing antibodies) as early as possible to reduce the risk of severe illness and death (Recommendation level: A; Evidence level: I).**

Lung cancer is a high-risk factor for severe illness and death from COVID-19 infection. Antiviral therapy should be used early in patients with lung cancer after COVID-19 infection, which can rapidly reduce the viral load and reduce the risk of severe illness and death. Antiviral small molecule drugs approved in China include Nirmatrelvir tablets/Ritonavir tablets, Simnotrelvir tablets/Ritonavir tablets, Azvudine, Molnupiravir capsules, and Deuremidevir hydrobromide tablets; antibody drug include BRII-196/BRII-198 monoclonal antibody.

During the anti-cancer treatment of patients with lung cancer, special attention should be paid to the interaction between chemotherapy drugs, targeted drugs, and antiviral drugs. Nirmatrelvir tablets/Ritonavir tablets should not be used with drugs that are highly dependent on CYP3A for clearance. The commonly used antitumor drugs and their interactions are shown in Table 1^[55]^. For patients with lung cancer who have moderate kidney injury, the dosage of Nirmatrelvir tablets/Ritonavir tablets should be reduced. Nirmatrelvir tablets/Ritonavir tablets should not be used in patients with severe kidney injury or severe liver injury.

**Table 1:** Interactions between Nirmatrelvir tablets/Ritonavir tablets (Paxlovid^®^) and anticancer drugs^[56]^

Anticancer drug	Nirmatrelvir tablet/Ritonavir tablets	Explanation
Almonertinib	Combination prohibited	A) The combination of 2 drugs affects the efficacy of Paxlovid^®^; B) The combination of the 2 drugs leads to high drug concentration causing severe adverse events; C) Discontinue, consider resuming the administration 3 days after the use of Paxlovid^®^.
Vometinib	Combination prohibited	
Gefitinib	Obvious interaction, preferably switch or discontinue	Discontinue or change to other alternative drugs. If necessary, pay close attention to the patient's symptoms and discontinue the drug in a timely manner.
Erlotinib	Obvious interaction, preferably switch or discontinue	
Afatinib	Obvious interaction, preferably switch or discontinue	
Savolitinib	Weak interaction, may be combined	Only partially metabolized by CYP3A4, weak interaction, low risk of severe adverse events. No action required.
Osimertinib	Can be used in combination	No interaction
Alectinib	Can be used in combination	
Pemetrexed	Can be used in combination	
Dacotinib	Can be used in combination	

Compared with antiviral small molecule drugs, COVID-19 neutralizing antibodies have no limitation on liver and kidney function and no limitation of concomitant medications, and are safer. The decision to administer neutralizing antibodies needs to be made in conjunction with the neutralizing activity of the main circulating strain. In addition to the BRII-196/BRII-198 monoclonal antibody approved in China, Tixagevimab/Cilgavimab has also been approved in Europe and Japan for the treatment of mild to moderate COVID-19 infection in patients over 12 years of age (body weight> 40 kg) with high risk factors. The results of the TACKLE study, a phase III clinical study, showed that treatment with Tixagevimab/Cilgavimab 600 mg intramuscularly within 7 days after the onset of symptoms of COVID-19 infection significantly reduced the risk of severe illness or death within 29 days by 50.5% compared with the placebo group (4.4% vs 8.9%)^[56]^.

### 6.2 Antitumor therapy

**Consensus 11: Patients with lung cancer who test positive for COVID-19 should consider to delay the antitumor treatment according to the severity of infection and tumor status; if the current condition is urgent and the tumor spread is uncontrolled, antitumor treatment is urgently needed, the severity of COVID-19 infection and the risk of tumor progression should be fully assessed, and the treatment should be continued under the guidance of professional physicians (Recommendation level: B; Evidence level: III).**

Radiation/chemotherapy may reduce the immune responses in patients with lung cancer, and the time to clear the virus may be prolonged in patients with lung cancer, leading to longer duration of the disease and poor prognosis. ICIs may reactivate depleted T cells, thus aggravating the inflammatory cytokine storm caused by COVID-19 and leading to organ damage. ICIs treatment can also cause adverse events such as immune pneumonia. If 3 kinds of diseases (immune pneumonia, lung cancer, and COVID-19) appear at the same time, clinical management will be difficult^[57]^. A large cohort study showed that recent chemotherapy treatment was identified as a predictor of severe COVID-19 infection, and the risk increased with the degree of myelosuppression (HR=17.31; 95%CI: 6.52- 45.98)^[58]^. Real-world data suggested that chemotherapy in combination with ICIs may be harmful in patients with COVID-19 infection^[59]^. In addition, there is a potential drug-drug interactions between antiviral and antitumor agents, and patients on long-term oral targeted antitumor agents are at high risk for severe drug-drug interactions. The United States NCCN recommended that for severe and critically ill patients with cancer, antitumor therapy should be delayed for >20 days if conditions allow, or until symptoms improve; for mild and moderate cases, it is recommended to delay treatment for >14 days, or until symptoms improve^[60]^. According to a survey of Chinese experts, when NSCLC is complicated with COVID-19 infection, 95.95% of the experts believe that chemotherapy should be discontinued, 86.91% of the experts believe that targeted therapy should be discontinued for severe/critical COVID-19, and 95.95% of the experts believe that immunotherapy should be discontinued^[61]^.

### 6.3 Treatment of traditional Chinese medicine

COVID-19 belongs to the category of epidemic disease in traditional Chinese medicine. Because of the feeling of “epidemic rage”, the disease can be treated according to the condition, syndrome, and climate. Qingfei Paidu decoction is suitable for mild, moderate, severe, and critical cases, and is used in accordance with the patient’s condition. Available Chinese patent medicines include Huoxiang Zhengqi capsules (soft capsules, pills, water, and oral liquid), Shufeng Jiedu capsules (granules), Qingfei Paidu granules, Huashi Baidu granules, Xuanfei Baidu granules, Sanhan Huashi granules, Jinhua Qinggan granules, and Lianhua Qingwen capsules (granules).

### 6.4 Management of patients with severe illness

Early identification of patients with severe illness is the key to the treatment of COVID-19. The National Health Commission issued the “Diagnosis and treatment for severe cases of COVID-19 infection (trial 4^th^ edition)”, proposing that for those who do not meet the diagnostic criteria for severe cases but have pneumonia caused by COVID-19 infection and have one of the following conditions can also be treated as severe cases: age >65 years, incomplete vaccination, complicated chronic diseases (including hypertension, diabetes, coronary heart disease, chronic lung disease, malignant tumors, immunocompromised, etc.)^[62]^. Therefore, patients with lung cancer should be managed as severe cases after infection with COVID-19, and early and active intervention should be performed to monitor vital sign measurements, especially finger oxygen saturation at rest and after activity, in the meanwhile monitoring the relevant indicators of the underlying disease. According to the “Diagnosis and treatment for COVID-19 infection (trial 10^th^ edition)”, the following changes should be alert to the deterioration of the disease: hypoxemia or progressive aggravation of respiratory distress; deterioration of tissue oxygenation indicators (eg, oxygen saturation, oxygenation index) or progressive increase of lactic acid; progressive decrease of peripheral blood lymphocyte count or progressive increase of inflammatory factors (eg, interleukin-6, C-reactive protein, ferritin); significant increase of coagulation function related indicators (eg, D-dimer); and significant progression of pulmonary lesions on chest imaging^[63]^.

In the treatment of severe cases, adequate energy and nutrition intake should be ensured, water and electrolyte balance should be paid attention to, and internal environment stability should be maintained. Those with high fever can undergo physical cooling and apply antipyretic drugs. Patients with severe cough and expectoration should be given anti-tussive and expectorant drugs. Patients should avoid blind or inappropriate use of antibiotics, especially in combination with broad-spectrum antibiotics. Those with underlying diseases should be treated accordingly. The main treatment measures for severe cases in the “Diagnosis and treatment for severe cases of COVID-19 infection (trial 4^th^ edition)” include antiviral therapy, immunotherapy, anticoagulant therapy, prone position therapy, oxygen therapy and respiratory support, circulation monitoring and support, nutritional support therapy, analgesia and sedation, acute kidney injury and kidney replacement therapy, and traditional Chinese medicine treatment^[62]^.

## 7 Conclusion

The main goal of epidemic prevention and control at the present stage in China is to “protecting people’s health and preventing severe cases”. Lung cancer is the most common tumor in China, patients with lung cancer are at high risk of severe illness and death caused by COVID-19 infection. The prevention and treatment of COVID-19 infection in patients with lung cancer should be highly concerned. COVID-19 vaccine is one of the most important therapeutic means to combat the COVID-19 epidemic. Patients with lung cancer should complete the full immunization and the booster immunization as early as possible. In addition, patients with lung cancer are a vulnerable population and the immune response rate and neutralizing antibody titers of these patients after vaccination are lower than those of healthy people. Neutralizing antibodies (Tixagevimab/Cilgavimab) can reduce the risk of COVID-19 infection, severe illness, and death, and are an important addition to protect patients with lung cancer. As the virus continues to mutate and the future epidemic trend is unpredictable, physicians, disease control personnel, and patients should pay attention to the new variant strain in real time, and pay attention to the progress of the research and development of COVID-19 vaccines and drugs, to maximize the benefit of patients with lung cancer during the epidemic.

## Acknowledgment

We thank Wenzhou YU from Center of Immunization Program, Chinese Center for Disease Control and Prevention, for contributing to this consensus.

## Competing interests

The authors declare no competing interests.

**Table:** 

Consultant Experts (listed in surname stroke order)	
Jinming YU	Shandong Cancer Hospital and Institute
Jie WANG	Cancer Hospital Chinese Academy of Medical Sciences
Hongzhou LU	The Third People's Hospital of Shenzhen
Ligang XING	Shandong Cancer Hospital and Institute
Weimin LI	West China Hospital of Sichuan University
Junling LI	Cancer Hospital Chinese Academy of Medical Sciences
Nong YANG	Hunan Cancer Hospital
Jianxing HE	The First Affiliated Hospital of Guangzhou Medical University
Yong SONG	Jinling Hospital, Medical School of Nanjing University
Li ZHANG	Sun Yat-sen University Cancer Center
Lizhu LIN	The First Affiliated Hospital of Guangzhou University of Traditional Chinese Medicine
Min ZHOU	Ruijin Hospital, Shanghai Jiaotong University School of Medicine
Caicun ZHOU	Shanghai Pulmonary Hospital, Tongji University School of Medicine
Qinghua ZHOU	West China Hospital of Sichuan University
Hua ZHONG	Shanghai Pulmonary Hospital
Shuanghu YUAN	Shandong Cancer Hospital and Institute

## References

[1] Xia C, Dong X, Li H, et al. Cancer statistics in China and United States, 2022: profiles, trends, and determinants. Chin Med J (Engl), 2022, 135(5): 584-590. doi: 10.1097/CM9.0000000000002108 35143424 PMC8920425

[2] Shiels MS, Haque AT, González AB, et al. Leading causes of death in the US during the COVID-19 pandemic, March 2020 to October 2021. JAMA Intern Med, 2022, 182(8): 883-886. doi: 10.1001/jamainternmed.2022.2476 35788262 PMC9257676

[3] Oldani S, Petrelli F, Dognini G, et al. COVID-19 and lung cancer survival: An updated systematic review and meta-analysis. Cancers (Basel), 2022, 14(22): 5706. doi: 10.3390/cancers14225706 36428798 PMC9688481

[4] World Health Organization. Coronavirus disease (COVID-19) pandemic. https://www.who.int/.https://www.who.int/ Accessed on 2023-01-20

[5] Liang X, Liu MW, Zhang L, et al. Global trends of incidence of lung cancer. Zhongguo Zhong Liu, 2022, 31(9): 683-692. doi: 10.11735/j.issn.1004-0242.2022.09.A002

[6] Wang Q, Berger NA, Xu R, et al. Analyses of risk, racial disparity, and outcomes among us patients with cancer and COVID-19 infection. JAMA Oncol, 2021, 7(2): 220-227. doi: 10.1001/jamaoncol.2020.6178 33300956 PMC7729584

[7] Dai M, Li D, Liu M, et al. Patients with cancer appear more vulnerable to SARS-CoV-2: A multicenter study during the COVID-19 outbreak. Cancer Discov, 2020 10(6): 783-791. doi: 10.1158/2159-8290.CD-20-0422 32345594 PMC7309152

[8] Luo J, Rizvi H, Preeshagul IR, et al. COVID-19 in patients with lung cancer. Ann Oncol, 2020, 31(10): 1386-1396. doi: 10.1016/j.annonc.2020.06.007 32561401 PMC7297689

[9] National Health Commission of the People’s Republic of China. Technical guidelines for vaccination of COVID-19 (version 1). Zhonghua Lin Chuang Gan Ran Bing Za Zhi, 2021, 14(2): 89-90. doi: 10.3760/cma.j.issn1674-2397.2021.02.002

[10] Chinese Society Oncological Supportive Care, Chinese Society Clinical Chemotherapy Committee. Chinese expert consensus on issues related to the protection, treatment and management of patients with solid tumors during COVID-19 (2022 edition). Zhonghua Zhong Liu Za Zhi, 2022, 44(10): 1083-1090. doi: 10.3760/cma.j.cn112152-20220505-00309 36319453

[11] Iriagac Y, Cavdar E, Karaboyun K, et al. Immunogenicity and safety of the CoronaVac vaccine in patients undergoing treatment for breast and lung cancer. Bratisl Lek Listy, 2022, 123(6): 401-407. doi: 10.4149/BLL_2022_063 35576541

[12] Ariamanesh M, Porouhan P, PeyroShabany B, et al. Immunogenicity and safety of the inactivated SARS-CoV-2 vaccine (BBIBP-CorV) in patients with malignancy. Cancer Invest, 2022, 40(1): 26-34. doi: 10.1080/07357907.2021.1992420 34634986 PMC8567287

[13] Paternina-Caicedo A, Jit M, Alvis-Guzmán N, et al. Effectiveness of CoronaVac and BNT162b 2 COVID-19 mass vaccination in Colombia: A population-based cohort study. Lancet Reg Health Am, 2022, 12: 100296. doi: 10.1016/j.lana.2022.100296 35791428 PMC9246705

[14] Simsek M, Yasin AI, Besiroglu M, et al. The efficacy of BNT162b 2 (Pfizer-BioNTech) and CoronaVac vaccines in patients with cancer. J Med Virol, 2022, 94(9): 4138-4143. doi: 10.1002/jmv.27835 35513241 PMC9348062

[15] Bestvina CM, Whisenant JG, Torri V, et al. Coronavirus disease 2019 outcomes, patient vaccination status, and cancer-related delays during the Omicron wave: A brief report from the TERAVOLT analysis. JTO Clin Res Rep, 2022, 3(8): 100335. doi: 10.1016/j.jtocrr.2022.100335 35619644 PMC9119707

[16] Ma Y, Zhu P, Zhong G, et al. Serial negative response after standard and third (Booster) dose of COVID-19 inactivated vaccine is associated with low vitamin D levels in patients with solid cancers. Front Med (Lausanne), 2022, 9: 898606. doi: 10.3389/fmed.2022.898606 35966864 PMC9373038

[17] Di Noia V, Pimpinelli F, Renna D, et al. Duration of humoral response to the third dose of BNT162b 2 vaccine in patients with solid cancer: Is fourth dose urgently needed? Eur J Cancer, 2022, 176: 164-167. doi: 10.1016/j.ejca.2022.09.006 36223679 PMC9548024

[18] National Comprehensive Cancer Network. Cancer and COVID-19 vaccination, version 7.0. https://www.nccn.org/docs/default-source/covid-19/2021_covid-19_vaccination_guidance_v7-0.pdf?sfvrsn=b483da2b_119.https://www.nccn.org/docs/default-source/covid-19/2021_covid-19_vaccination_guidance_v7-0.pdf?sfvrsn=b483da2b_119 Accessed on 2022-09-22

[19] Joint Prevention and Control Mechanism of the State Council, National Health Commission of the People’s Republic of China. Work plan for strengthening the vaccination of the elderly against COVID-19. http://www/nhc.gov.cn/xcs/gzzcwj/202211/9bb71c9c7d664fb0bbcd2b3eaaefcf84.shtml.http://www/nhc.gov.cn/xcs/gzzcwj/202211/9bb71c9c7d664fb0bbcd2b3eaaefcf84.shtml Accessed on 2023-01-20

[20] Joint Prevention and Control Mechanism of the State Council. Notice on issuing the implementation plan for the second booster vaccination of COVID-19. http://www.gov.cn/xinwen/2022-12/14/content_5731889.htm.http://www.gov.cn/xinwen/2022-12/14/content_5731889.htm Accessed on 2023-01-20

[21] Bobrovitz N, Ware H, Ma X, et al. Protective effectiveness of previous SARS-CoV-2 infection and hybrid immunity against the omicron variant and severe disease: a systematic review and meta-regression. Lancet Infect Dis, 2023. doi: 10.1016/S1473-3099(22)00801-5 PMC10014083 36681084

[22] Wang Z, Muecksch F, Schaefer-Babajew D, et al. A Naturally enhanced neutralizing breadth against SARS-CoV-2 one year after infection. Nature, 2021, 595(7867): 426-431. doi: 10.1038/s41586-021-03696-9 34126625 PMC8277577

[23] COVIDSurg Collaborative, GlobalSurg Collaborative. SARS-CoV-2 vaccination modelling for safe surgery to save lives: data from an international prospective cohort study. Br J Surg, 2021, 108(9): 1056-1063. doi: 10.1093/bjs/znab101 33761533 PMC7995808

[24] Yasin AI, Aydin SG, Sümbül B, et al. Efficacy and safety profile of COVID-19 vaccine in cancer patients: a prospective, multicenter cohort study. Future Oncol, 2022, 18(10): 1235-1244. doi: 10.2217/fon-2021-1248 35081732 PMC8793921

[25] Tang K, Wei Z, Wu X. Impaired serological response to COVID-19 vaccination following anticancer therapy: A systematic review and meta-analysis. J Med Virol, 2022, 94(10): 4860-4868. doi: 10.1002/jmv.27956 35750492 PMC9349696

[26] Yuan J, Tan XH, Wang FX, et al. Expert advice on novel coronavirus vaccination in patients with malignant tumors. Xin Fa Chuan Ran Bing Dian Zi Za Zhi, 2022, 7(2): 1-5. doi: 10.19871/j.cnki.xfcrbzz.2022.02.001

[27] Shinada K, Murakami S, Yoshida D, et al. Radiation recall pneumonitis after COVID-19 vaccination. Thorac Cancer, 2022, 13(1): 144-145. doi: 10.1111/1759-7714.14239 34791816 PMC8652508

[28] Ma Y, Liu N, Wang Y, et al. Immune checkpoint blocking impact and nomogram prediction of COVID-19 inactivated vaccine seroconversion in patients with cancer: a propensity-score matched analysis. J Immunother Cancer, 2021, 9(11): e003712. doi: 10.1136/jitc-2021-003712 PMC8634011 34845005

[29] Mei Q, Hu G, Yang Y, et al. Impact of COVID-19 vaccination on the use of PD-1 inhibitor in treating patients with cancer: a real-world study. J Immunother Cancer, 2022, 10(3): e004157. doi: 10.1136/jitc-2021-004157 PMC8915379 35264438

[30] Pezeshki PS, Rezaei N. Immune checkpoint inhibition in COVID-19: risks and benefits. Expert Opin Biol Ther, 21(9): 1173-1179. doi: 10.1080/14712598.2021.1887131 PMC7898453 33543652

[31] Song Q, Bates B, Shao YR, et al. Risk and outcome of breakthrough COVID-19 Infections in vaccinated patients with cancer: real-world evidence from the National COVID Cohort Collaborative. J Clin Oncol, 2022, 40(13): 1414-1427. doi: 10.1200/JCO.21.02419 35286152 PMC9061155

[32] Valanparambil RM, Carlisle J, Linderman SL, et al. Antibody response to COVID-19 mRNA vaccine in patients with lung cancer after primary immunization and booster: reactivity to the SARS-CoV-2 WT virus and Omicron variant. J Clin Oncol, 2022, 40(33): 3808-3816. doi: 10.1200/JCO.21.02986 35759727 PMC9671759

[33] Lee LYW, Lonescu MC, Starkey T, et al. COVID-19: Third dose booster vaccine effectiveness against breakthrough coronavirus infection, hospitalisations and death in patients with cancer: a population-based study. Eur J Cancer, 2022, 175: 1-10. doi: 10.1016/j.ejca.2022.06.038 36084618 PMC9276646

[34] Dong J, Zost SJ, Greaney AJ, et al. Genetic and structural basis for SARS-CoV-2 variant neutralization by a two-antibody cocktail. Nat Microbiol, 2021, 6(10): 1233-1244. doi: 10.1038/s41564-021-00972-2 34548634 PMC8543371

[35] Levin MJ, Ustianowski A, De Wit S, et al. Intramuscular AZD. N Engl J Med, 2022, 386(23): 2188-2200. doi: 10.1056/NEJMoa2116620 35443106 PMC9069994

[36] Alhumaid S, Mutair AA, Alali J, et al. Efficacy and safety of Tixagevimab/Cilgavimab to prevent COVID-19 (pre-exposure prophylaxis): a systematic review and meta-analysis. Diseases, 2022, 10(4): 118. doi: 10.3390/diseases10040118 36547204 PMC9777759

[37] Suribhatla R, Starkey T, Ionescu MC, et al. Systematic review of the clinical effectiveness of Tixagevimab/Cilgavimab for prophylaxis of COVID-19 in immunocompromised patients. medRxiv. 2022. https://www.medrxiv.org/content/10.1101/2022.11.07.22281786v1.https://www.medrxiv.org/content/10.1101/2022.11.07.22281786v1 Accessed on 2023-01-20. doi: 10.1101/2022.11.07.22281786 37006158

[38] Young-Xu Y, Epstein L, Marconi VC, et al. Tixagevimab/Cilgavimab for rrevention of COVID-19 during the Omicron surge: retrospective analysis of national VA electronic data. medRxiv. 2022. https://www.medrxiv.org/content/10.1101/2022.05.28.22275716v1.https://www.medrxiv.org/content/10.1101/2022.05.28.22275716v1 Accessed on 2023-01-20. doi: 10.1101/2022.05.28.22275716 PMC10470809 37535398

[39] Chen B, Haste N, Binkin N, et al. Real world effectiveness of Tixagevimab/cilgavimab (Evusheld) in the Omicron era. medRxiv. 2022. https://www.medrxiv.org/content/10.1101/2022.09.16.22280034v1.https://www.medrxiv.org/content/10.1101/2022.09.16.22280034v1 Accessed on 2023-01-20. doi: 10.1101/2022.09.16.22280034 PMC10138227 37104498

[40] Highlights of Emergency Use Authorization (EUA). Fact sheet for healthcare providers: emergency use authorization for Evusheld^TM^ (tixagevimab copackaged with cilgavimab). http://www.fda.gov/media/154701/download.http://www.fda.gov/media/154701/download Accessed on 2023-01-20

[41] National Comprehensive Cancer Network Cancer and COVID-19 vaccination, version 7.0. http://www.nccn.org/covid-19http://www.nccn.org/covid-19 Accessed on 2023-01-20

[42] Kertes J, Shapiro Ben David S, Engel-Zohar N, et al. Association between AZD 7442 (Tixagevimab-Cilgavimab) administration and severe acute respiratory syndrome coronavirus 2 (SARS-CoV-2) infection, hospitalization, and mortality. Clin Infect Dis, 2023, 76(3): e126-e132. doi: 10.1093/cid/ciac625 35904210 PMC9384583

[43] Nguyen Y, Flahault A, Chavarot N, et al. Pre-exposure prophylaxis with tixagevimab and cilgavimab (Evusheld) for COVID-19 among 1112 severely immunocompromised patients. Clin Microbiol Infect, 2022, 28(12): 1654. e1-1654.e4. doi: 10.1016/j.cmi.2022.07.015 PMC9340091 35926762

[44] National Health Commission of the People’s Republic of China. Diagnosis and treatment plan for COVID-19 (trial version 9). Zhonghua Lin Chuang Gan Ran Bing Za Zhi, 2022, 15(2): 81-89. doi: 10.3760/cma.j.issn.1674-2397.2022.02.001

[45] Young RP, Hopkins R J, Christmas T, et al. COPD prevalence is increased in lung cancer, independent of age, sex and smoking history. Eur Respir J, 2009, 34(2): 380-386. doi: 10.1183/09031936.00144208 19196816

[46] Hashimoto N, Matsuzaki A, Okada Y, et al. Clinical impact of prevalence and severity of COPD on the decision-making process for therapeutic management of lung cancer patients. BMC Pulm Med, 2014, 14: 14. doi: 10.1186/1471-2466-14-14 24498965 PMC3922111

[47] Li J, Zheng J, Yuan J, et al. Protective effect of a bacterial extract against acute exacerbation in patients with chronic bronchitis accompanied by chronic obstructive pulmonary disease. Chin Med J (Engl), 2004, 117(6): 828-834. 15198881

[48] Collet JP, Shapiro P, Ernst P, et al. Effects of an immunostimulating agent on acute exacerbations and hospitalizations in patients with chronic obstructive pulmonary disease. The PARI-IS Study Steering Committee and Research Group. Prevention of acute respiratory infection by an immunostimulant. Am J Respir Crit Care Med, 1997, 156(6): 1719-1724. doi: 10.1164/ajrccm.156.6.9612096 9412546

[49] Pan L, Jiang XG, Guo J, et al. Effects of OM-85 BV in patients with chronic obstructive pulmonary disease: a systematic review and meta-analysis. J Clin Pharmacol, 2015, 55(10): 1086-1092. doi: 10.1002/jcph.518 25903441

[50] Bacteriological and Viral Cooperation Group of Changchun Institute of Biopharmaceutical Research. Progress of vaccine for prevention and treatment of chronic bronchitis. Yi Xue Yan Jiu Tong Xun, 1972(4): 20-22.

[51] Kearney SC, Dziekiewicz M, Feleszko W. Immunoregulatory and immunostimulatory responses of bacterial lysates in respiratory infections and asthma. Ann Allergy Asthma Immunol, 2015, 114(5): 364-369. doi: 10.1016/j.anai.2015.02.008 25752734

[52] Liu XB, Li F, Zheng XY. The effects of long-term prophylactic dose of Staphylococcus and Neisseria Tablets on immune function and life quality in patients with moderately severe COPD patients. Wenzhou Yi Ke Da Xue Xue Bao, 2018, 48(9): 676-679, 683.

[53] Shehadeh F, Benitez G, Mylona EK, et al. A pilot trial of thymalfasin (thymosin-α-1) to treat hospitalized patients with hypoxemia and lymphocytopenia due to coronavirus disease 2019 infection. J Infect Dis, 2023, 227(2): 226-235. doi: 10.1093/infdis/jiac362 36056913 PMC9494344

[54] Sun LD, Deng LH, Jiang Y, et al. Expert consensus on personal protection of melanoma patients during novel coronavirus infected pneumonia (first edition). Xian Dai Zhong Liu Yi Xue, 2020, 28(8): 1412-1414. doi: 10.3969/j.issn.1672-4992.2020.08.043

[55] Chinese Society of General Practice, China Association of Traditional Chinese Medicine Branch of General Practice, Respiratory Disease Prevention and Control Speciality Society of Chinese Preventive Medicine Association, et al. Guideline for diagnosis, treatment and management of COVID-19 in primary care (first edition). Zhonghua Quan Ke Yi Shi Za Zhi, 2023, 22(2): 115-137. doi: 10.3760/cma.j.cn114798-20230108-00040

[56] Montgomery H, Hobbs FDR, Padilla F, et al. Efficacy and safety of intramuscular administration of tixagevimab-cilgavimab for early outpatient treatment of COVID-19 (TACKLE): a phase 3, randomised, double-blind, placebo-controlled trial. Lancet Respir Med, 2022, 10(10): 985-996. doi: 10.1016/S2213-2600(22)00180-1 35688164 PMC9173721

[57] Pezeshki PS, Rezaei N. Immune checkpoint inhibition in COVID-19: risks and benefits. Expert Opin Biol Ther, 2021, 21(9): 1173-1179. doi: 10.1080/14712598.2021.1887131 33543652 PMC7898453

[58] Clift AK, Coupland CAC, Keogh RH, et al. Living risk prediction algorithm (QCOVID) for risk of hospital admission and mortality from coronavirus 19 in adults: national derivation and validation cohort study. BMJ, 2020, 371: m3731. doi: 10.1136/bmj.m3731 33082154 PMC7574532

[59] Moubarak S, Merheb D, Basbous L, et al. COVID-19 and lung cancer: update on the latest screening, diagnosis, management and challenges. J Int Med Res, 2022, 50(9): 3000605221125047. doi: 10.1177/03000605221125047 PMC9515530 36154328

[60] National Comprehensive Cancer Network®. Prevention and treatment of cancer-related infections (version 3, 2022), NCCN clinical practice guidelines in oncology. https//www.nccn.org/professionals/physician_gls/pdf/infections.pdf.https//www.nccn.org/professionals/physician_gls/pdf/infections.pdf Accessed on 2023-01-20

[61] Liu M, Zhao W, Li SY, et al. Current status of diagnosis and treatment of advanced non-small cell lung cancer in China during the COVID-19 pandemic. Ann Palliat Med, 2022, 11(4): 1231-1240. doi: 10.21037/apm-21-72 34894701

[62] The National Health Commission, State Administration of Traditional Chinese Medicine Department. Diagnosis and treatment for severe cases of COVID-19 infection (trial 4^th^ edition). http://www.nhc.gov.cn/xcs/zhengcwj/202301/ad34a9b598654b90a5c8c99440cce21b.shtml.http://www.nhc.gov.cn/xcs/zhengcwj/202301/ad34a9b598654b90a5c8c99440cce21b.shtml Accessed on 2023-01-20

[63] The National Health Commission, State Administration of Traditional Chinese Medicine Department. Diagnosis and treatment for COVID-19 infection (trial 10^th^ Edition). http://www.nhc.gov.cn/xcs/zhengcwj/202301/32de5b2ff9bf4eaa88e75bdf7223a65a.shtml.http://www.nhc.gov.cn/xcs/zhengcwj/202301/32de5b2ff9bf4eaa88e75bdf7223a65a.shtml Accessed on 2023-01-20

